# Management of refractory intramural left ventricular summit ventricular arrhythmia: Acute success using bipolar radiofrequency catheter ablation with recurrence

**DOI:** 10.1002/joa3.12937

**Published:** 2023-10-05

**Authors:** Koji Sudo, Kenji Kuroki, Tetsuya Asakawa, Kazutaka Aonuma, Akira Sato

**Affiliations:** ^1^ Department of Cardiovascular Medicine University of Yamanashi Chuo Japan; ^2^ Department of Cardiology Yamanashi Kosei Hospital Yamanashi Japan; ^3^ Department of Cardiology Mito Saiseikai General Hospital Mito Japan

**Keywords:** bipolar radiofrequency catheter ablation, over‐the‐wire microelectrodes catheter, ventricular premature contraction

## Abstract

Bipolar radiofrequency catheter ablation (RFCA) is a novel strategy for refractory or recurrent ventricular arrhythmias (VAs) resistant to conventional ablation methods. Lesions created during bipolar RFCA are larger than those created during sequential unipolar ablation. We present a case of refractory LV summit VAs, which identified the origin using a 2.7‐F over‐the‐wire microelectrodes catheter, and it was successfully treated with bipolar RFCA in the acute phase.

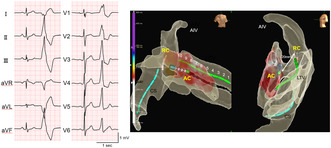

Radiofrequency catheter ablation (RFCA) is an effective treatment for ventricular arrhythmias (VAs). The success rate depends on the VA origin, with difficulties in RFCA occurring when VAs originate in the intramural myocardium or epicardium.

Bipolar RFCA is a novel treatment strategy for refractory or recurrent VAs resistant to conventional ablation methods. Lesions created during bipolar RFCA are larger than those created during sequential unipolar ablation.^1^ Here, we present a case, in which the origin of refractory left ventricular (LV) summit VA was identified using a 2.7‐F over‐the‐wire microelectrodes catheter and it was successfully treated with bipolar RFCA in the acute phase.

A 25‐year‐old male patient was referred to our hospital for catheter ablation (CA) to treat symptomatic and refractory ventricular premature contractions (VPCs) and non‐sustained ventricular tachycardia (VT). The patient had a history of frequent VPCs and non‐sustained VT and had undergone endocardial ablation of the LV at a different hospital 8 years prior. It was difficult to eliminate VPCs and non‐sustained VT. After the procedure, the patient received flecainide and a β‐blocker, sotalol, and follow‐up examination was continued for years. Echocardiography revealed a normal ejection fraction. A total of 19 909 VPCs (21%) were documented with 24‐h Holter monitoring before the second ablation procedure. A 12‐lead electrocardiogram (ECG) showed clinical VPCs, which exhibited an inferior axis, right bundle branch block, and a maximum deflection index of 0.6, suggesting an epicardial origin (Figure 1A).

**FIGURE 1:**
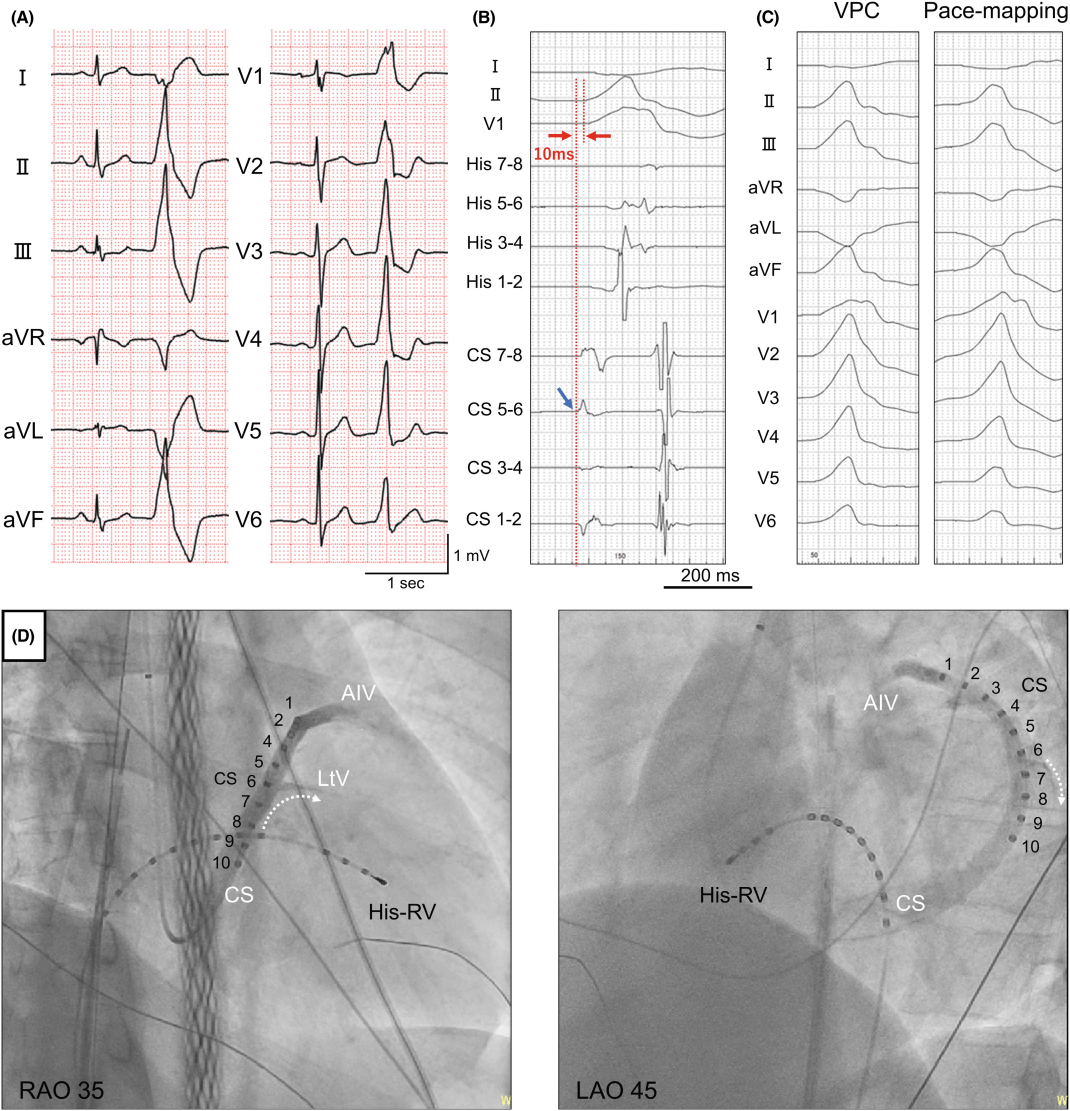
(A) 12‐lead ECG. The 12‐lead ECG shows a sinus rhythm and ventricular premature complex (second beat), right bundle branch block, and an inferior axis. (B) The earliest site (CS5‐6) of the 10‐electrode catheter (*blue arrow*), compared to distal CS electrodes, preceded the QRS onset by only 10 ms (*red arrows*). (C) Pace‐mapping from the LV lateral endocardium was not good, compared to clinical VPC. (D) Fluoroscopic views and CS venography. White dotted arrows show the LtV. AIV, anterior interventricular vein; CS, coronary sinus; ECG, electrocardiogram; LAO, left anterior oblique; LtV, lateral vein; RAO, right anterior oblique; RV, right ventricle.

The patient provided informed consent for the procedure, and an electrophysiological study was performed. After coronary sinus (CS) venography and coronary artery angiography, the earliest site (CS5‐6) of the 10‐electrode catheter placed in the CS preceded the QRS onset by only 10 ms (Figure 1B). Pace mapping from the LV lateral endocardium was not good (Figure 1C), and CS venography showed a lateral vein (Figure 1D). Therefore, a 0.014‐inch guide wire was inserted into the lateral vein, and a 2.7‐F over‐the‐wire microelectrodes catheter (0.65‐mm electrode size with 5.0‐mm interelectrode spacing; EPstar Fixed AIV; Japan Lifeline) was smoothly inserted into the lateral vein along the guide wire (Figure 2A). The bipolar electrograms of the lateral vein ostium preceded the more distal one with the LtV7‐8 and LtV9‐10 electrodes preceding the VPC‐QRS onset by 20 ms (Figure 2B), and pace‐mapping at the LtV7‐8 electrode showed good pace matching to the clinical VPC (Figure 2C). After an intracardiac echocardiography‐guided transseptal puncture, a 3.5‐mm irrigated‐tip contact force‐sensing ablation catheter (TactiCath Sensor Enabled; Abbott Laboratories) was placed at the opposing LV endocardial site. However, the local bipolar electrograms during VPCs at the ablation catheter preceded VPC‐QRS onset by only 5 ms, and pace‐mapping at the site did not show good matching as previously described. Radiofrequency (RF) energy between 35 and 40 W was delivered with a contact force of 10–15 ***g*** and a targeted lesion size index of 6.0–7.0. After the endocardial RF applications, VPCs disappeared only transiently. Unipolar RF application in the ostium of the LV summit was impossible due to high impedance.

**FIGURE 2:**
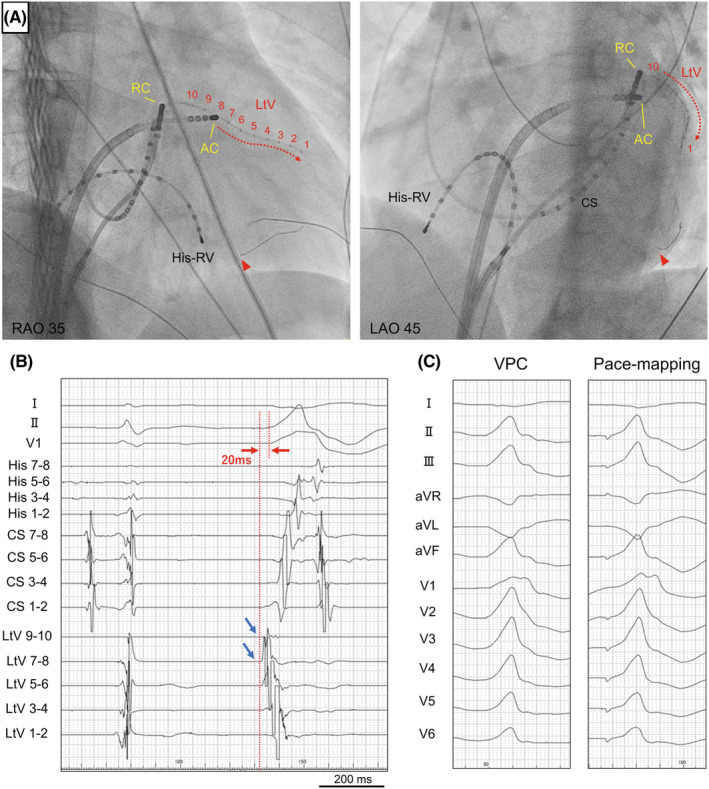
(A) Fluoroscopic views (RAO 35/LAO 45) and the over‐the‐wire microelectrodes catheter position into the lateral vein (*red dotted arrows*) and the catheters during bipolar radiofrequency ablation between the left ventricular endocardial site (ablation catheter; TactiCath) and ostium of the LV summit (return catheter; Bernoulli). A 0.014‐inch guidewire through an inner lumen was inserted into the LtV and shows the distal site of the guidewire (*red arrow head*). (B) The bipolar electrograms of the LtV ostium preceded the more distal one with the LtV7‐8 and LtV9‐10 electrodes (*blue arrow*) preceding the VPC‐QRS onset by 20 ms (*red arrows*). (C) Pace‐mapping at the LtV7‐8 electrode showed good pace matching to the clinical VPC. AC, active catheter; ECG, electrocardiogram; LAO, left anterior oblique; LtV, lateral vein; RAO, right anterior oblique; RC, return catheter; RV, right ventricle.

Therefore, bipolar RFCA was subsequently performed. The TactiCath was still located at the opposing LV endocardial site as an active catheter (AC), and a bidirectional 4.0‐mm open‐irrigated catheter (Ablaze Bernoulli 7F; Japan Lifeline Co.) was placed at the ostium of the LV summit as a return catheter (RC) (Figure 2A). An RC irrigation continued during bipolar RFCA at an irrigation rate of 10 mL/min. Bipolar RFCA was performed using RF energy (a power: 20–30 W, mean power: 23 W), with the AC at the LV endocardial site and the RC at the ostium of the LV summit. A three‐dimensional mapping system (EnSite X; Abbott Laboratories) shows activation mapping of the LV endocardium, and the catheter distance between the AC and RC was 20 mm (Figure 3A). The coronary angiogram confirmed that the ablation site was not in proximity to the coronary arteries. The initial average impedance was 177 Ω, and impedance was carefully monitored during bipolar RFCA. When impedance increased or decreased by more than 20 Ω, ablation was instantly stopped. The maximal RC temperature was also recorded carefully to avoid exceeding 70°C during bipolar RFCA. The first bipolar RF application led to the successful elimination of the VPC (Figure 3B). During a 20‐min waiting period after the application, clinical VPC recurred in an infrequent form, we performed additional applications around the temporary successful site and reconfirmed the total elimination. During bipolar application, typical impedance, temperature, and power trends were observed (Figure 3C). Owing to the frequent temperature rise in the RC, bipolar RFCA applications were performed with different durations (duration: 20–120 s, mean duration: 58 s), and the total bipolar RF duration was 17.4 min with 19 applications.

**FIGURE 3:**
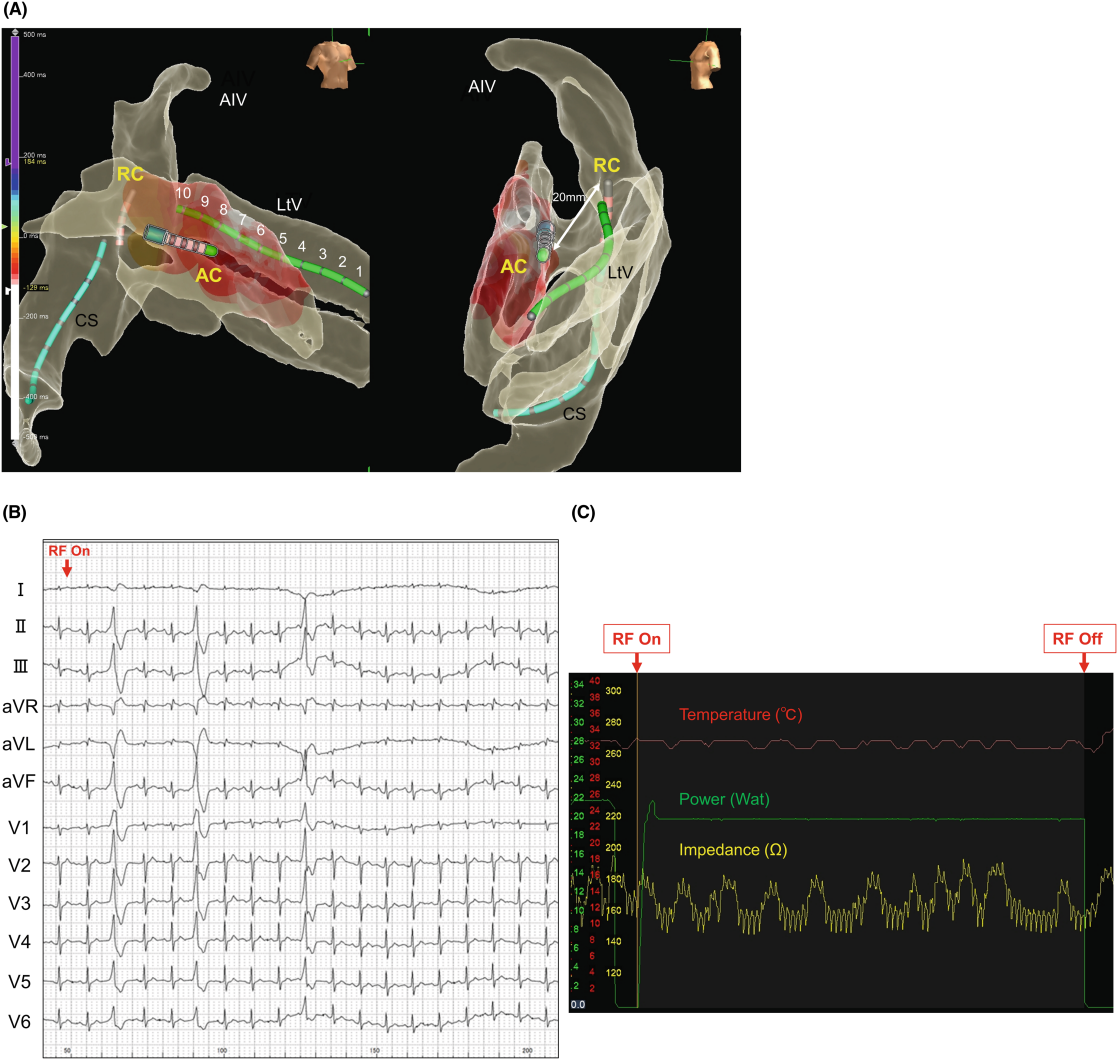
(A) EnSite image with RAO and LAO view shows an activation mapping and position of the active and return catheters. The ablation catheter distance was 20 mm in the three‐dimensional mapping. (B) A 12‐lead ECG showing bipolar RFCA application. VPCs were eliminated immediately after bipolar RFCA started. (C) Power, impedance, and temperature graph during bipolar ablation. AC, active catheter; CS, coronary sinus; ECG, electrocardiogram; LAO, left anterior oblique; LtV, lateral vein; RAO, right anterior oblique; RC, return catheter; RF, radiofrequency.

After the ablation procedure, isoproterenol and adenosine triphosphate were infused; however, no clinical VPC was induced. There were no complications, and VPCs were not noted during ECG monitoring at the hospital. The VPC burden was 40.7% in the 3‐month Holter ECG monitoring. However, all VPCs were isolated and no non‐sustained VT was documented, even though the patient discontinued sotalol and flecainide after the ablation procedure. This case was considered as acute success with recurrent LV summit VAs following bipolar RFCA.

CA of VAs to intramural myocardial origin is still a clinically important issue. In our case, the location of this VA was left basal lateral of the LV, and thus it originated from the inaccessible region of the LV summit. Various methods other than conventional CA have been devised to treat intramural VAs. Representative techniques such as ablation using half‐normal saline, needle ablation, and ethanol infusion of the target vessel have been reported.^2^ There are various bail‐out strategies for failed conventional unipolar RFCA, but at our hospital, bipolar RFCA is routinely chosen as the first bail‐out strategy because we are experienced in bipolar RFCA most of all.

In our case, we chose bipolar RFCA with a 3.5‐mm irrigated‐tip contact force‐sensing AC and a 4.0‐mm open‐irrigated catheter as guided by our clinical experience and supported by previous reports.^3^ Bipolar RFCA is a novel treatment strategy for refractory or recurrent VAs resistant to conventional ablation methods. However, there are few reports on the efficacy and safety of bipolar RFCA, especially when one of the catheters is inserted into the coronary venous system.^4^ Furthermore, in our case, we could have used a large 8.0‐mm‐tip catheter as the RC instead of a 4.0‐mm‐tip catheter, which could have helped to reduce the impedance of the bipolar RFCA. In our case, we attempted to clarify and address the limitations associated with bipolar RFCA, and we expect that bipolar RFCA will be increasingly used to successfully treat intramural VAs in the future. However, the optimal settings of bipolar RFCA, including details regarding ablation parameters such as wattage, application time, duration, and lesion size index, still need to be established. Moreover, the types of catheter tips to be used as the AC and RC require further clarification.

Igarashi et al.^5^ reported acute and long‐term results of bipolar RFCA for VAs of an intramural origin. Their study showed that bipolar RFCA is useful for acute suppression of refractory VT. However, they reported the use of bipolar RFCA between the coronary venous system and LV endocardium in only one patient. Therefore, it is important to evaluate the anatomy of the coronary venous system with CS venography. Furthermore, the VT recurrence rate was reported to be high, and the mortality rate during the follow‐up period was relatively low after coronary venous system ablation of VAs originating from the LV summit. In our case, all medications other than a β‐blocker were discontinued. Although VPC recurrence was observed after the blanking period, non‐sustained VT was not observed, contributing to the improvement of subjective symptoms. Nevertheless, the durability of bipolar RFCA remains to be verified. Additionally, a combination of bipolar RFCA with other treatments, such as the epicardial approach and chemical ablation, should be considered.

Several reports have described the complications of bipolar RFCA,^5^ of which the most important are pericardial effusion, atrioventricular block in patients with LV septum origin, and coronary artery stenosis in patients with LV summit origin. To ensure the safety of bipolar RFCA, it is necessary to consider changes in impedance and temperature during RF application.

The 2.7‐F over‐the‐wire microelectrodes catheter is useful to identify the origin of VAs that are situated close to the coronary venous system, but cannot conclusively identify the exact origin of the arrhythmia in all cases. Bipolar RFCA can reduce the severity of intramural LV summit VAs. However, there are still issues associated with bipolar RFCA, and another approach may be needed to treat challenging VAs.

## CONFLICT OF INTEREST STATEMENT

Kenji Kuroki and Akira Sato have received research grant and honoraria from Abbott Medical Japan, LLC. All other authors have no conflicts to disclose.

## PATIENT CONSENT STATEMENT

Patient consent for publication was obtained.

## References

[1] Koruth JS , Dukkipati S , Miller MA , Neuzil P , d'Avila A , Reddy VY . Bipolar irrigated radiofrequency ablation: a therapeutic option for refractory intramural atrial and ventricular tachycardia circuits. Heart Rhythm. 2012;9:1932–1941. 10.1016/j.hrthm.2012.08.001 22863684

[2] Neira V , Santangeli P , Futyma P , Sapp J , Valderrabano M , Garcia F , et al. Ablation strategies for intramural ventricular arrhythmias. Heart Rhythm. 2020;17:1176–1184. 10.1016/j.hrthm.2020.02.010 32087355

[3] Nagashima K , Watanabe I , Okumura Y , Ohkubo K , Kofune M , Ohya T , et al. Lesion formation by ventricular septal ablation with irrigated electrodes: comparison of bipolar and sequential unipolar ablation. Circ J. 2011;75:565–570. 10.1253/circj.CJ-10-0870 21187654

[4] Futyma P , Sander J , Ciąpała K , Głuszczyk R , Wysokińska A , Futyma M , et al. Bipolar radiofrequency ablation delivered from coronary veins and adjacent endocardium for treatment of refractory left ventricular summit arrhythmias. J Interv Card Electrophysiol. 2020;58:307–313. 10.1007/s10840-019-00609-9 31402415

[5] Igarashi M , Nogami A , Fukamizu S , Sekiguchi Y , Nitta J , Sakamoto N , et al. Acute and long‐term results of bipolar radiofrequency catheter ablation of refractory ventricular arrhythmias of deep intramural origin. Heart Rhythm. 2020;17:1500–1507. 10.1016/j.hrthm.2020.04.028 32353585

